# Assessment of energy loss across aortic valves using accelerated CMR multi-point flow measurements

**DOI:** 10.1186/1532-429X-14-S1-W9

**Published:** 2012-02-01

**Authors:** Christian Binter, Verena Knobloch, Robert Manka, Andreas Sigfridsson, Sebastian Kozerke

**Affiliations:** 1 Institute for Biomedical Engineering, University and ETH Zurich, Zurich, Switzerland

## Summary

A novel approach for evaluating the performance of artificial or diseased heart valves is presented and applied on in-vitro as well as in-vivo aortic valve data. The method, which is based on turbulence and flow measurements, provides a measure to assess and compare energy dissipation under varying flow conditions.

## Background

Diseased or artificial heart valves possibly lead to turbulent flow and regurgitation, both increasing the workload of the heart. Current measures for valve assessment, i.e. effective orifice area, only indirectly and partially correlate with the energy loss due to the valve [1]. Phase-Contrast MRI makes it possible to directly quantify these energy losses, and by relating them to kinetic energy of the flow a parameter describing the hemodynamic performance of the valve can be obtained.

## Methods

**Figure 1:**
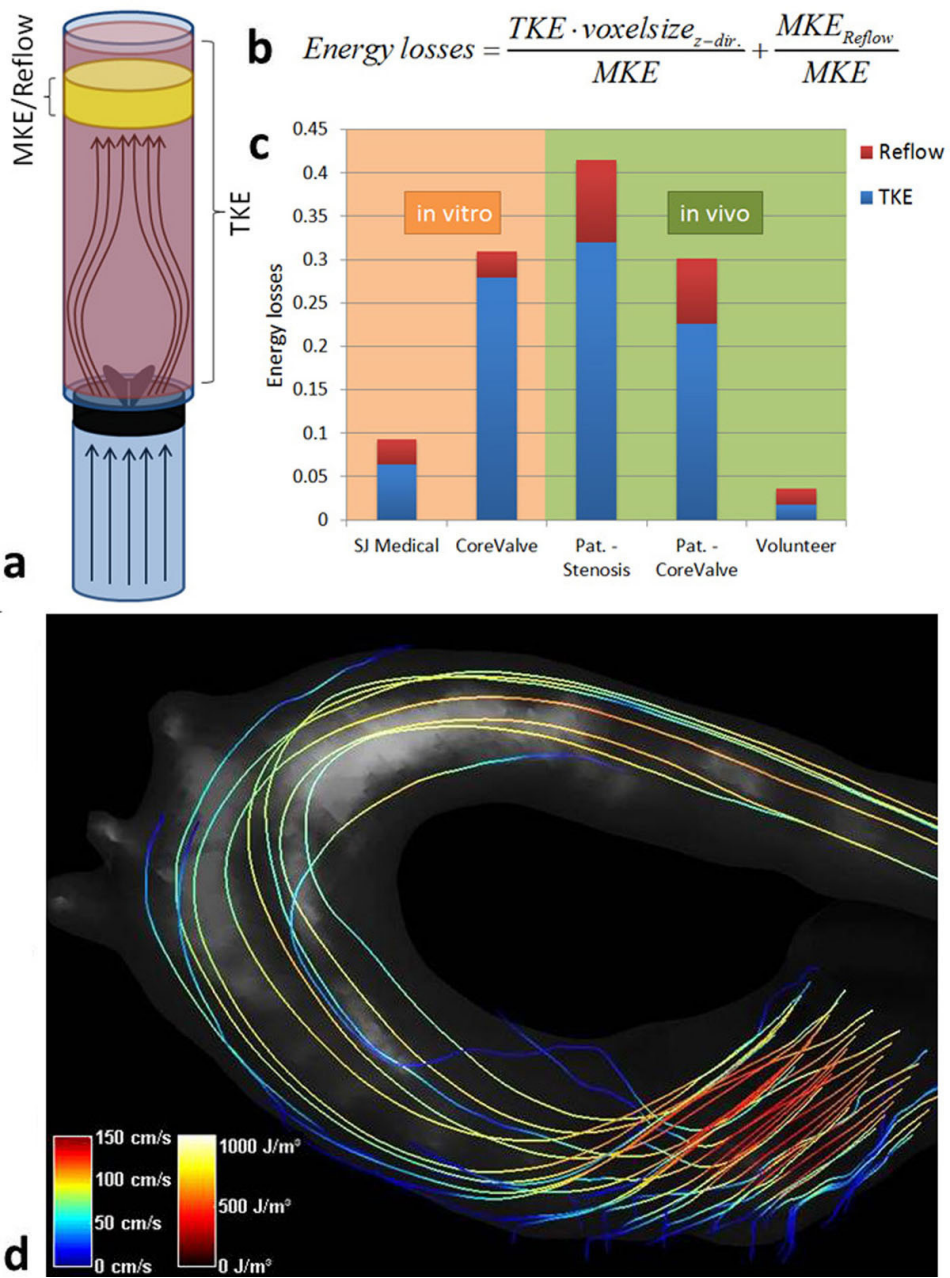
a) Phantom setup and regions where MKE and TKE are calculated. TKE is integrated over the whole volume. For MKE a region downstream from the valve is taken, in which flow is assumed laminar. In this region the slices in flow direction are averaged to obtain the values for a single slice. Regular and regurgitant flow are treated separately. b) Energy losses are calculated by relating TKE to MKE, normalized by the voxel size in flow direction, and by relating the regurgitant flow to the forward flow. c) The results of in vitro and in vivo testing. The orifice areas of the two valves tested in vitro differ, possibly leading to a more pronounced disparity. d) Streamlines and TKE in a healthy volunteer during systole. Same scaling as in Fig. 2, no TKE isosurfaces visible because all values are below the threshold.

3D Phase-Contrast flow measurements of the aortic arch employing multiple first gradient moments provide not only information about velocities, but also allow conclusions about the turbulence intensities over a large dynamic range. Measurements with 3 different encoding steps in each direction were combined using a Bayesian analysis method adapted from [2] to estimate the 4D velocity vector field and turbulent kinetic energy (TKE) as proposed in [3]. The TKE was taken and set into relation to the mean kinetic energy (MKE) of the flow. Additionally, the MKE of the forward and the regurgitant flow were compared to yield a more complete picture of possible energy losses (Fig. 1a-b).

In-vitro measurements were performed using a home-built pulsatile flow phantom to study a mechanical St. Jude Medical standard bileaflet valve (St. Jude Medical Inc., St. Paul, MN, USA), as well as a biological Transcatheter Medtronic CoreValve (Medtronic Inc., Minneapolis, MN, USA). In vivo data were acquired in 6 healthy volunteers as well as in two patients with a stenotic valve (valve area 0.9 cm^2^, mean gradient 34 mmHg) and a Medtronic CoreValve, respectively.

All data were acquired on a 3T Achieva system (Philips Healthcare, Best, The Netherlands) with cardiac triggering and navigator gating. The voxel size was 2 mm isotropic, and temporal resolution was 34 ms. Employing 8-fold undersampling and k-t PCA reconstruction, the nominal scan time was about 8 min without navigator efficiency taken into account.

## Results

**Figure 2:**
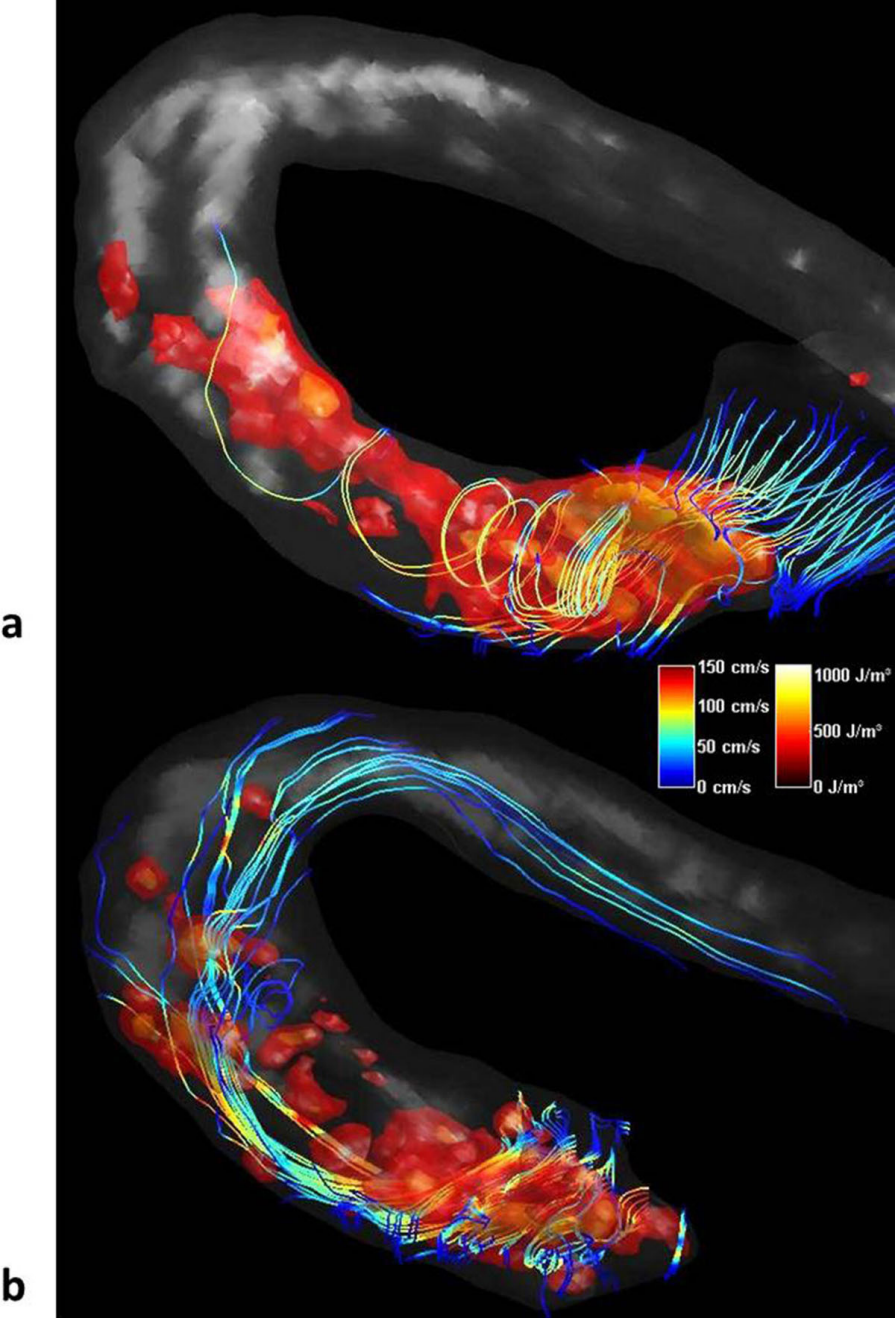
Complex flow patterns during systole in the ascending aorta of two patients, a) with a diseased aortic valve, and b) with a CoreValve implanted 2 years before measurement. The colors of the streamlines correspond to velocities, and the isosurfaces to different levels of TKE. Because of the signal void inside the CoreValve (b) only the distal part of the flow is displayed.

Fig. 1c shows the relative energy losses due to turbulence and regurgitation in-vitro and in-vivo. The streamline visualization of the flow as well as an isosurface rendering of TKE values in both patients can be seen in Fig. 2. The maximum TKE values were about 150 J/m^3^ in volunteers, and 950 J/m^3^ respectively 540 J/m^3^ in the patients with the stenotic and the artificial valve. Patient stroke volumes were 68 ml and 80 ml, respectively.

## Conclusions

A method for assessing valve performance independent of flow rates has been proposed. It has been demonstrated that relative energy loss differs across heart valve designs and values have been found to be 6-fold higher compared to normal subjects. The work indicates that quantification of relative energy losses may provide a potential parameter to characterize the efficiency of the cardiovascular system in general and vascular prostheses in particular.
